# Supplementary material for: Aging-Affected MSC Functions and Severity of Periodontal Tissue Destruction in a Ligature-Induced Mouse Periodontitis Model
Source: Int J Mol Sci. 2020 Oct 30;21(21):8103. doi: 10.3390/ijms21218103 (PMC7663404; doi:10.3390/ijms21218103)
Supplement: Supplementary file 1 [file ijms-21-08103-s001.pdf]

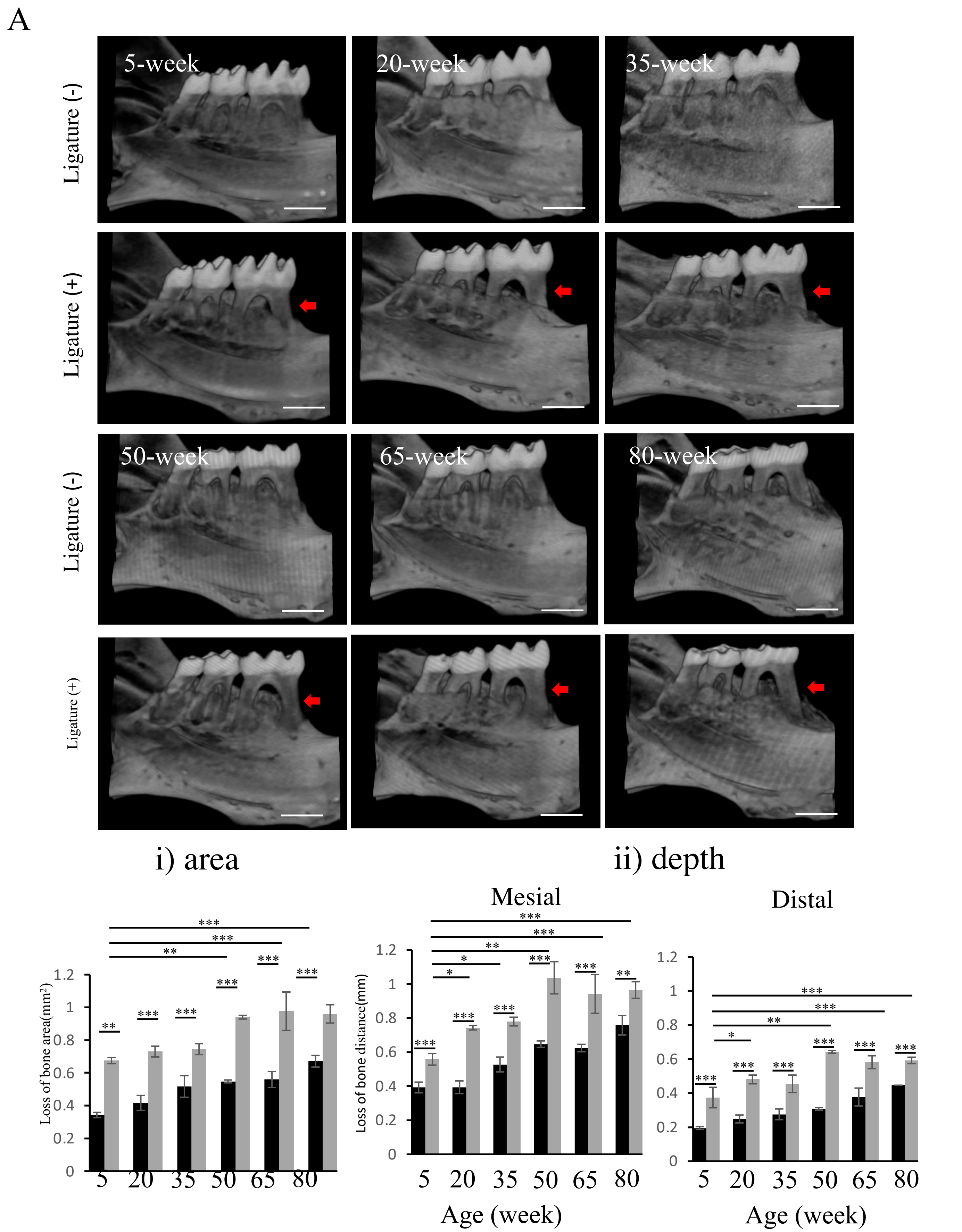

Experimental induced periodontitis in different age mice. (A) Micro-CT images of control and periodontitis model of 5,20,35, 50, 65 and 80-week old mice at Day-10 after ligation. Bar:100 mm. (B) (i) Area and (ii) Depth measurement data show the aged group(50,65,80 week) mice show more severe bone loss compared to young (5,20,35 week)mice. There was significant interaction between age and ligation. Data are presented as mean± SEM (n=3, two-way ANOVA *$p < 0.05$, **$p < 0.01$, ***$p < 0.001$)

Aung et al., Supplymentary Fig. S1

A

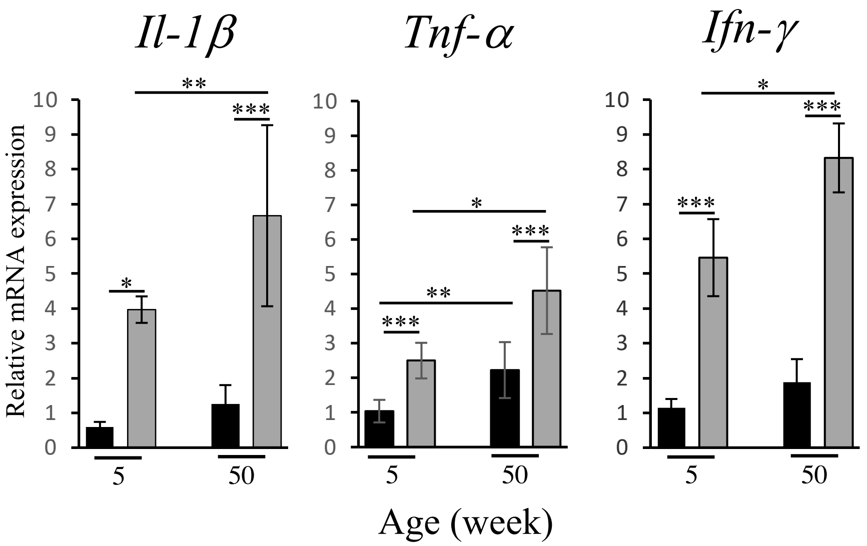

Inflammatory cytokine levels were increased in aged periodontitis model mice. Relative expression of inflammatory cytokines from first molar area of 5- and 50-week old mice. Elevated expression of *Tnf-α, Il-1β, Inf-γ* was observed in 50-week old mice compared to 5-week old mice at day-10 after ligation. There was significant interaction between age and ligation in *Il-1β.* Data are presented as mean± SEM (n=3, two-way ANOVA, Tukey test .*$p < 0.05$, **$p < 0.01$, ***$p < 0.001$)

A

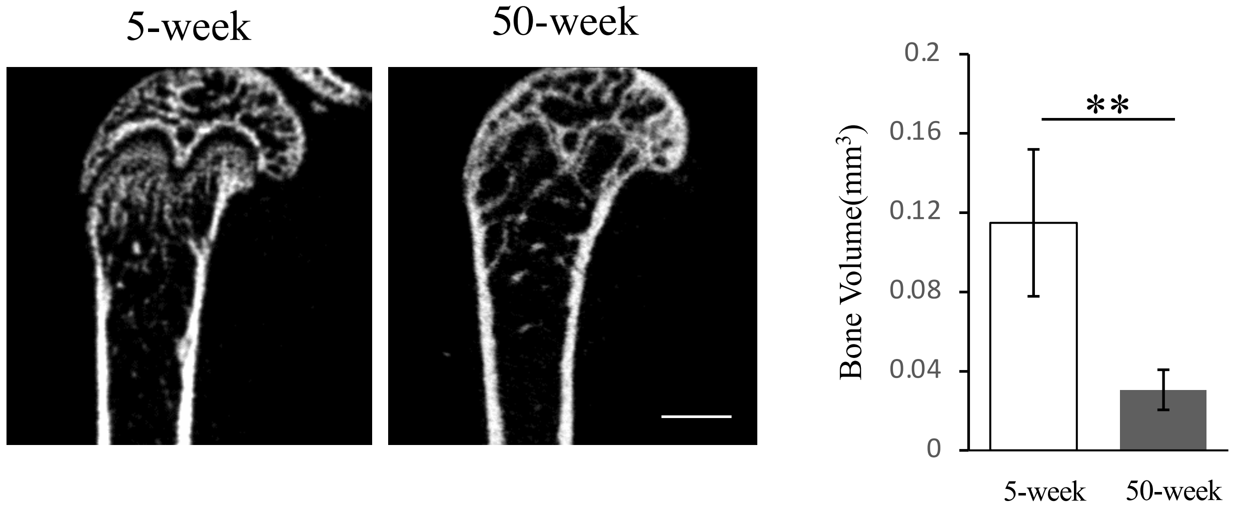

Aged mice showed decreased bone volume. (**A**) Micro CT analysis showed decreased bone volume in 50-week old mice compared to 5-week old mice (unpaired Students' t-test , ***p* < 0.01).
